# Development of Machine Learning Models for Estimating Metabolizable Protein Supply from Feed in Lactating Dairy Cows

**DOI:** 10.3390/ani15050687

**Published:** 2025-02-26

**Authors:** Mingyung Lee, Dong Hyeon Kim, Seongwon Seo, Luis O. Tedeschi

**Affiliations:** 1 Department of Animal Science, Texas A&M University, College Station, TX 77843, USA; mingyung@tamu.edu; 2 Dairy Science Division, National Institute of Animal Science, Rural Development Administration, Cheonan 31000, Republic of Korea; kimdh3465@korea.kr; 3 Division of Animal and Dairy Sciences, Chungnam National University, Daejeon 34134, Republic of Korea; swseo@cnu.ac.kr

**Keywords:** duodenal microbial nitrogen, lactating Holstein cows, random forest regression, rumen-undegradable protein, support vector regression

## Abstract

Accurate prediction of protein utilization in dairy cows is essential for optimizing nutrition and milk yield to achieve sustainable cattle production. This study aimed to develop novel machine learning models to predict rumen-undegradable protein (RUP) and duodenal microbial nitrogen (MicN) based on dietary protein intake. A dataset comprising 1779 observations from 436 scientific publications was used to train support vector regression (SVR) and random forest regression (RFR) models. Different predictor sets were identified for each model, including factors such as days in milk (DIM), dry matter intake (DMI), dietary fiber content, and crude protein fractions. Model performance was evaluated using statistical metrics, including the coefficient of determination (R^2^), root mean square error of prediction (RMSEP), and concordance correlation coefficient (CCC), with results compared to existing NASEM (2021) models. The RFR model provided the most precise and unbiased predictions for RUP (R^2^ = 0.60, RMSEP = 0.326 kg/d, CCC = 0.71), while the SVR model was most effective for MicN (R^2^ = 0.76, RMSEP = 42.4 g/d, CCC = 0.86). Both models outperformed traditional methods, demonstrating the potential of machine learning in improving protein utilization predictions. Future studies could explore hybrid approaches integrating conventional and AI-based models to enhance predictive accuracy.

## Simple Summary

Predicting how much protein from the diet is digested and used by dairy cows is crucial for improving their nutrition and milk yield while achieving sustainable cattle production. This study used novel machine learning techniques to develop models that predict two important protein-related factors: rumen-undegradable protein (RUP) and duodenal microbial nitrogen (MicN). By analyzing a large dataset from scientific studies, we created two models using support vector regression (SVR) and random forest regression (RFR). The results showed that our models outperformed traditional prediction methods, providing more accurate and precise estimates. The RFR model was the best for predicting RUP, while the SVR model performed best for MicN. These improvements could help farmers and animal nutritionists formulate better diets for dairy cows, reducing waste and improving efficiency. Future research could combine traditional and machine learning-based models to refine predictions further, benefiting the dairy industry and the environment.

## 1. Introduction

Nutritional management of lactating dairy cows is a critical aspect of dairy production, directly influencing milk yield, animal health, and overall farm profitability and long-term environmental sustainability [1,2]. Among the various nutrients, dietary protein is an important precursor for milk protein synthesis and a supporter of essential metabolic and physiological functions [3,4]. Metabolizable protein (MP), which is defined as the total digestible and absorbable nitrogen sources (digestible total amino acids) in the small intestine, is a key factor determining protein availability for dairy cows [5,6]. MP supply primarily originates from rumen-undegradable protein (RUP) and microbial nitrogen (MicN). Therefore, accurate prediction of MP supply hinges on precise estimates of these components, which remain challenging due to the intricate and dynamic interactions among feed digestion, ruminal fermentation, and post-ruminal absorption [7,8]. These challenges are further compounded by the variable composition of the ruminal microbial population and its stochastic behavior, particularly in the processes of attachment to and fermentation of feed particles within the ever-changing ruminal environment [9,10]. Proper MP nutrition is not only essential for meeting the cow’s protein requirements but also plays a pivotal role in maintaining microbial balance and overall rumen efficiency [11,12]. Optimizing rumen health through targeted nutritional strategies enhances microbial functionality, supports fiber digestion, and mitigates risks associated with ruminal acidosis and other digestive disorders. Since metabolizable protein plays a pivotal role in maintaining microbial balance and rumen efficiency, its accurate prediction is crucial for sustainable and efficient dairy production [13].

Traditional mechanistic models have been employed to estimate MP supply (RUP and MicN) based on the chemical composition of feed ingredients and total digestible nutrients [5,6]. While these models provide valuable insights, their reliance on predefined equations and static parameters limits their ability to account for biological systems’ inherently nonlinear and multifactorial nature. In contrast, machine learning has emerged as a potential alternative to traditional modeling approaches in the dairy research sector [14,15]. Machine learning regression algorithms are effective at analyzing large, complex datasets and identifying intricate patterns [16,17,18], which suggests they may be particularly appropriate for applications such as predicting biological outcomes, including MP supply. In the dairy field, machine learning algorithms have been mainly used to solve classification problems [17], such as disease diagnosis (i.e., lameness and mastitis) or calving detection [19,20,21,22,23,24], but they are also being used in regression problems such as predicting milk yield and feed intake [25,26,27]. By integrating diverse datasets, including feed composition, animal performance metrics, and environmental variables, machine learning-based models hold the potential to significantly improve prediction accuracy and adaptability in dairy cow nutrition management.

This study develops and evaluates machine learning-based sub-models that predict the key components, RUP and MicN, to estimate the MP supply in lactating dairy cows. By leveraging advanced machine learning regression techniques, this research seeks to address the limitations of traditional models, enhance our understanding of protein metabolism, and provide practical, data-driven tools for optimizing protein feeding strategies. Ultimately, the findings aim to support greater economic efficiency and reduce the environmental footprint of dairy production. This work represents a foundational step toward developing hybrid intelligent mechanistic models (HIMMs; [14,15,28]) that combine the predictive power of machine learning with the biological insight of mechanistic modeling approaches.

## 2. Materials and Methods

### 2.1. Database Construction and Dataset Extraction

The database used in this study was an integrated dataset created by merging two published and peer-reviewed databases, containing data exclusively from lactating Holstein cows. The final dataset consisted of 1779 observations from 436 publications. The first source was a collection of papers published in the Animal Nutrition section of the *Journal of Dairy Science* between 2016 and 2020, which contributed to 1326 observations from 319 articles that had been previously described by Jeon et al. [29]. The second source was the modeling database provided by the National Animal Nutrition Program (https://animalnutrition.org; accessed on 17 October 2023) in the United States, encompassing data published from 1979 to 2017 and contributing to 453 observations from 117 articles. Descriptive statistics for the integrated database are presented in Table 1. A thorough verification process ensured that there were no duplicate articles between the two databases, confirming the integrity of the combined dataset.

The MP was defined as the sum of RUP and MicN, following the definitions provided by NASEM [5]. To ensure comparability, predictions were aligned with the outputs of the NASEM model. Observed RUP (kg/d) was calculated as the observed duodenal flows of non-ammonia, non-microbial nitrogen (NANMN, g/d) minus the estimated endogenous nitrogen (N) flows, with the nitrogen values multiplied by 6.25 to convert them to crude protein (CP). Endogenous N flows (g/d) were estimated using the equation defined by NASEM: 15.4 + 1.21 × DMI (kg/d). The NASEM RUP model was employed as follows:

Dt_RUP = Σ[(1 − DCa_c_) × (Dt_CPAIn_c_ − Dt_CPAIn_npn,c_) + (Kp_c_/(Kp_c_ + Kd_c_)) ×
 Dt_CPBIn_c_ + Dt_CPCIn_c_], for c = 1 to N_c_ (1)

where Dt_RUP is the total diet concentration of the specified nutrient, c represents the feed class, ${DCa}_{c}$ is the ruminal degradability of fraction A (6.4%) in feed class c, ${Dt\_CPAIn}_{c}$ is the CP intake of fraction A within feed class c, ${Dt\_CPAIn}_{npn,c}$ represents the intake of ruminally degraded nonprotein nitrogen (NPN) converted to CP equivalents within feed class c, ${Dt\_CPBIn}_{c}$ is the intake of crude protein of fraction B within feed class c, ${Dt\_CPCIn}_{c}$ is the CP intake of fraction C within feed class c, ${Kp}_{c}$ is the static rate of passage for feed class c (e.g., 4.87%/h for forage or 5.2%/h for concentrate), and ${Kd}_{c}$ is the weighted average of the in situ degradation rate for each feed in class c.

The MicN was determined using the following equation:

Microbial N (g/d) = [101 + (82.6 × RDP)]/[(1 + 0.094/RDNDF) × (1 + 0.027/RDS)](2)

where RDP is rumen-degradable protein.

The rumen-degraded neutral detergent fiber (RDNDF) was calculated as

RDNDF = [−31.9 + (0.721 × NDF) − (0.247 × St) + (6.63 × CP) − (0.211 × CP^2^) (38.7 × ADF/NDF) − (0.121 × ForWet) + (1.51 × DMI) × ((NDF/100) × DMI)]/100(2a)

and rumen-degraded starch (RDS) was calculated as

RDS = [(71.2 − (1.45 × DMI) + (0.424 × fNDF) + (1.39 × St) − (0.0219 × St^2^) − (0.154 × ForWet)) × (St/100) × DMI]/100(2b)

where RDNDF represents rumen-degraded neutral detergent fiber, RDS represents rumen-degraded starch, ForWet is the concentration of wet forage in the diet (greater than 20% DM), DMI is dry matter intake (kg/d), St is starch, and CP, ADF, and fNDF are expressed as a percentage of dry matter.

The following were selected as the candidate explanatory variables for RUP (kg/d) and MicN (g/d): the animal information (i.e., days in milk [DIM], parity, body weight [BW, kg], dry matter intake [DMI, kg/d], milk yield [MY, kg/d]) and the dietary components (% DM) (i.e., dry matter [DM, % as-fed], organic matter [OM], CP, neutral detergent insoluble fiber [NDF], acidic detergent insoluble fiber [ADF], non-starch carbohydrate [NSC], fat, ash, starch). The CP intake calculated for each protein fraction (kg/d) using the NASEM [5] feed library was also included as a candidate variable. Milk composition, total tract digestibility, rumen characteristic data, and outcome data post-feed digestion were excluded from candidate variables. Separate datasets containing RUP and MicN, along with the corresponding candidate variables, were extracted for subsequent modeling and are described in Table 2 and Table 3.

### 2.2. Model Development and Model Evaluation

**Figure 1:**
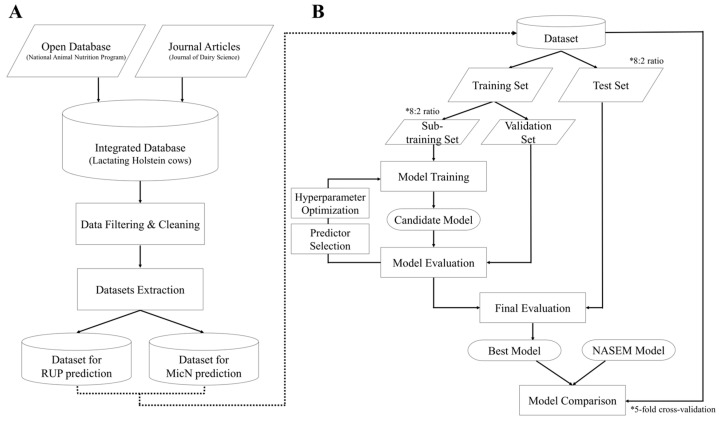
Flowchart of dataset preparation (**A**) and model development and evaluation processes (**B**).

Variables with limited quantities were excluded from the dataset to predict RUP and MicN. The refined dataset was split into training (80%) and test (20%) sets. The training set was further divided into sub-training (80%) and validation (20%) sets to determine the optimal combination of input variables and hyperparameters. The optimal input variables and hyperparameters were selected based on the model that achieved the highest precision and accuracy when evaluated on the validation set. The final optimal model was then retrained using the entire training set and evaluated on the test set. Subsequently, a new dataset containing only the input variables selected for the final model was extracted from the original dataset. A 5-fold cross-validation was conducted using this dataset to assess the model’s robustness. Under the same evaluation conditions, the NASEM [5] models (Equations (1) and (2)) were also evaluated using the 5-fold cross-validation method for a direct performance comparison. The entire process of dataset preparation and modeling is illustrated in Figure 1.

Both support vector regression (SVR) and random forest regression (RFR) techniques were employed to predict RUP and MicN. SVR, an application of support vector machine algorithm, utilized the Gaussian radial basis function (RBF) kernel, which involves two hyperparameters: gamma and cost [30,31]. The optimal gamma and cost were determined via a grid search within the 10-fold cross-validation framework using the tune function in the e1071 package of R. RFR, an application of the random forest algorithm that is widely applied in both classification and regression problems [32], involved two hyperparameters: the number of trees (*ntree*) and the number of variables considered at each split (*mtry*). These hyperparameters were also optimized through grid search, and modeling was performed using the randomForest package in R.

Model adequacy was evaluated based on several statistical measures outlined by Tedeschi [33]. The precision and accuracy of all models were assessed using the coefficient of determination (R^2^), root mean square error of prediction (RMSEP), and concordance correlation coefficient (CCC). CCC values were categorized following Hinkle et al. (1988) as negligible (0.00–0.30), low (0.30–0.50), moderate (0.50–0.70), high (0.70–0.90), and very high (0.90–1.00). Residual analyses were conducted to evaluate mean and slope biases. All statistical analyses and modeling were performed using R software (version 4.3.1). Statistical significance was determined at *p* < 0.05, while trends were noted for 0.05 ≤ *p* < 0.1.

## 3. Results

### 3.1. RUP Prediction

In the dataset extracted for RUP prediction, variables with insufficient quantities were excluded to ensure the robustness of the model development process (i.e., dietary organic matter (OM), fat, ash, non-structural carbohydrate (NSC), and starch contents), such as when the number of variables themselves was small (less than 20% of the dataset size, in this case, fewer than 100) or when the number of data points where they coexisted with other variables was small (fewer than 100). A total of 147 treatment means containing the remaining variables after exclusion (parity, BW, DIM, DMI, CP intake, CP fraction (A, B, and C) intake, dietary DM, NDF, ADF, and CP) were utilized for model development. Of these, 117 treatments (80% of the dataset) were allocated to the training set to select optimal input variables and hyperparameters. During the training process, the optimal input variable combinations for the RUP prediction model were identified as DIM, DMI, dietary DM content, CP B fraction intake, and CP C fraction intake. For the selection of optimal hyperparameters, the SVR model achieved the best performance with gamma set to 1 and cost set to 5, while the RFR model performed optimally with 100 *mtree* and 2 *mtry*. These models were subsequently evaluated using a test set (n = 30), and the RFR model exhibited better precision (R^2^ = 0.64) and accuracy (RMSEP = 0.35 kg/d) than the SVR model (Table 4).

**Figure 2:**
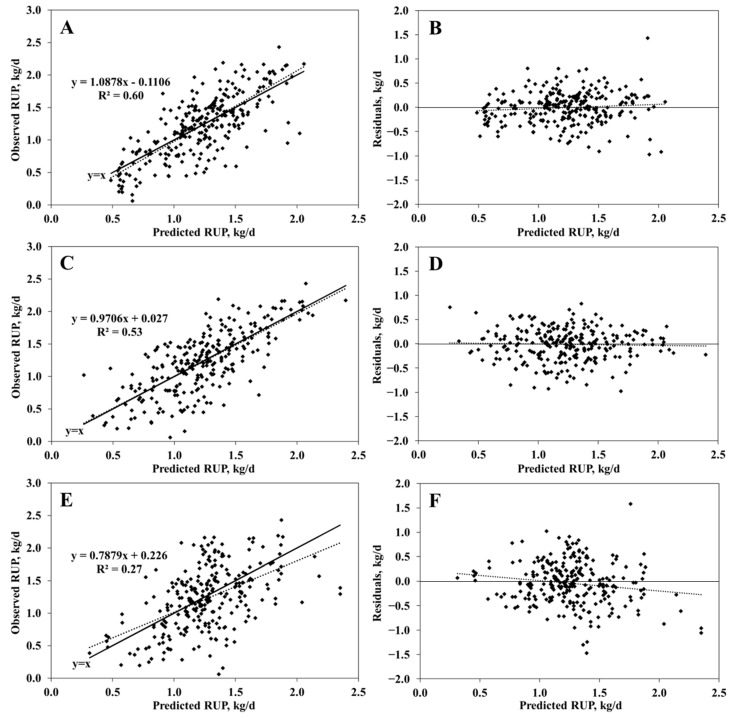
Plots of the observed (upper) and residual values (observed minus predicted values, lower) versus the model-predicted rumen-undegraded protein (RUP, kg/d). (**A**,**B**) are plots of a model developed with a random forest regression algorithm. (**C**,**D**) are plots of a model developed with a support vector regression algorithm. (**E**,**F**) are plots of the NASEM (2021) model.

To address the limited size of the initial dataset (n = 147), the dataset was re-extracted using the selected variable combinations to increase the sample size, resulting in 261 samples. The model adequacy was then re-evaluated using this re-sampled dataset, employing the equation (Equation (1)) from NASEM [5] (Table 5 and Figure 2). The new models explained 60% and 53% of the variation in observed RUP for the RFR and SVR models, respectively, which was approximately twice the explanatory power of the NASEM [5] model (Equation (1)). No statistically significant bias was observed in any of the evaluated models (*p* > 0.10). The RMSEP and CCC values for the RFR model, which was the best-performing model, were 0.33 kg/d and 0.71, respectively.

### 3.2. MicN Prediction

As in the RUP model development process, variables with insufficient quantities, as mentioned above, were excluded, and the excluded variables were the same type as those removed during RUP model development. A total of 154 treatment means containing the remaining variables after exclusion (parity, BW, DIM, DMI, CP intake, CP fraction (A, B, and C) intake, dietary DM, NDF, ADF, and CP) were used for model development. Optimal variables and hyperparameters were identified using a training set of 123 treatments (80% of the dataset), with the best combination including DIM, DMI, dietary DM and NDF contents, and CP A, B, and C fraction intakes. The optimal hyperparameters were determined to be gamma = 0.1 and cost = 15 for the SVR model, and *mtree* = 150 and *mtry* = 2 for the RFR model. The models were subsequently evaluated on a test set of 31 samples, where the SVR model demonstrated superior precision (R^2^ = 0.89) and accuracy (RMSEP = 38.6 g/d) compared to the RFR model (Table 6).

**Figure 3:**
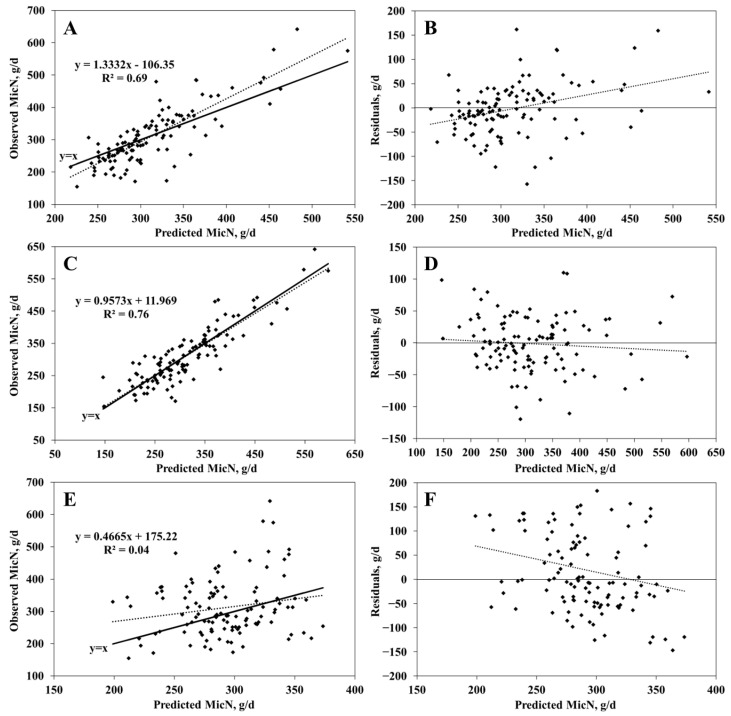
Plots of the observed (**A**,**C**,**E**) and residual values (observed minus predicted values; (**B**,**D**,**F**) versus the model-predicted duodenal microbial nitrogen (MicN, g/d). (**A**,**B**) are plots of a model developed with random forest regression algorithm. (**C**,**D**) are plots of a model developed with support vector regression algorithm. (**E**,**F**) are plots of the NASEM (2021) model.

The SVR model explained 89% of the variation in observed microbial nitrogen without any mean or slope bias (*p* > 0.10). The RMSEP and CCC values for the SVR model were 38.6 g/d and 0.94, respectively. However, the RFR model also exhibited high adequacy (R^2^ = 0.82 and RMSEP = 49.8 g/d) without any mean and slope biases. Unlike the RUP equation (Equation (1)) from NASEM [5], the MicN dataset lacked sufficient data for rumen-degraded NDF and starch. A separate dataset of 113 samples was extracted for comparison with the NASEM [5] equations (Equation (2)). Model adequacy was again evaluated using 5-fold cross-validation (Table 7 and Figure 3), and the SVR model demonstrated the highest precision and accuracy (R^2^ = 0.76 and RMSEP = 42.4 g/d). The RFR model exhibited significant slope bias in certain folds, while the NASEM [5] model showed lower accuracy and precision.

## 4. Discussion

Accurately estimating the flow of RUP and MicN to the duodenum is essential for precisely feeding dairy cows [7,8,34]. Although various models have been developed to predict these flows, many have demonstrated low accuracy or structural biases [5,6,7,8]. These limitations stem from the complex interrelationships among the variables used and the diverse factors contributing to transforming dietary protein sources into RUP and MicN, including animal and microbial influences. Therefore, this study aimed to overcome the limitations of previous models by leveraging machine learning techniques while utilizing the input variables traditionally employed in existing approaches.

This study identified five variables (i.e., DIM, DMI, dietary DM content, CP B, and CP C fraction intake) as optimal predictors for RUP estimation. Similarly, for MicN estimation, the optimal predictors were DIM, DMI, dietary DM, NDF contents, and CP A, B, and C fraction intake. Both machine learning models showed improved performance when days in milk and dry matter intake were included as input variables. In general, dairy cows undergo physiological changes in milk yield, body weight, and feed intake across different stages of lactation, and these changes can influence ruminal protein dynamics [35,36]. In particular, variations in DMI during this period directly affect the amount of CP supplied to the rumen, highlighting its importance as a key variable for predicting RUP and MicN. In previous studies, DIM has primarily been utilized to predict factors such as intake and body weight [37,38,39]. However, findings from this study suggest that DIM may also serve as an important variable in nutritional models. While DIM solely represents the number of days since parturition, it can act as a temporal marker that aligns with lactation-related changes in metabolism and nutrient utilization, which may not be fully accounted for by DMI alone. DMI represents actual feed intake, whereas DIM provides information on the lactation stage, which is associated with physiological adjustments influencing protein flow in the rumen [40]. Given these differences, DIM could provide complementary predictive value beyond what is captured by DMI alone. Moreover, DIM and DMI exhibit a nonlinear relationship, where DMI typically decreases after parturition, then gradually increases before declining again in later lactation stages [37]. This dynamic pattern suggests that using DMI alone may not fully account for changes in protein utilization over the lactation cycle. By incorporating DIM, models may be able to capture stage-dependent metabolic variations, potentially improving the prediction of RUP and MicN. Machine learning approaches, capable of capturing complex, nonlinear relationships and interactions among input variables, could leverage both DIM and DMI to enhance predictive accuracy. In our study, the inclusion of DIM improved model performance, suggesting that it contributes meaningful additional information. Given these advantages, incorporating DIM into machine learning models has the potential to improve model generalizability across different lactation stages and contribute to more accurate predictions.

Dietary DM content and CP B and C fraction intakes were also selected as input variables in both models. These variables are directly associated with the quantity of protein supplied to the rumen and the ruminal degradation rates, likely contributing to the improved model performance compared to other variables, as adopted by the NASEM [5] model. In the MicN prediction model, dietary NDF content was also included, which may be attributed to its significant influence on ruminal microbial growth and ruminal passage rates, as noted in both the NASEM and CNCPS systems [5,41].

The machine learning-based models developed in this study for predicting RUP and MicN demonstrated superior precision and accuracy compared to the NASEM [5] model. This enhanced performance can be attributed to the capacity of machine learning methods to incorporate multiple variables while effectively managing multicollinearity [42,43]. Furthermore, machine learning algorithms excel at capturing the complexity and nonlinear relationships between input and output variables, which makes them more suitable than conventional regression models for modeling ruminal protein outflow in dairy cows. Previous research has mentioned the nonlinearity among variables in dairy cow modeling, and studies have successfully applied machine learning regression algorithms to predict milk production, feed intake, and nitrogen excretion [25,27,44,45,46]. These findings underscore the potential of machine learning techniques in advancing the prediction of ruminal protein flow. In this study, the NASEM [5] model demonstrated significantly lower precision and accuracy in predicting MicN than the developed machine learning models. While the coefficient of determination (R^2^) was not reported in NASEM [5], the root mean square error of prediction (RMSEP) was found to be 83 g/d, which is consistent with the 90.7 g/d reported in this study. To ensure the validity of these results, we conducted multiple tests using the NASEM [5] model and its official software, and the performance remained unchanged.

The RFR model demonstrated superior accuracy and precision in RUP prediction compared to the SVR model; however, statistically significant mean and slope biases were observed. Similarly, for MicN prediction, the RFR model exhibited a statistically significant slope bias, which was not present in the SVR model. Both algorithms are recognized for performing well with relatively small datasets [47,48], but differences in their design and data processing approaches may explain these outcomes. The RFR model combines multiple decision trees, partitioning data nonlinearly to capture local patterns [32,49]. This ensemble approach provides flexibility and is well suited for modeling complex, nonlinear relationships; however, its reliance on discrete tree splits may hinder its ability to efficiently capture smooth linear trends [50], potentially contributing to the biases observed in our study.

In contrast, the SVR model optimizes a hyperplane across the entire data space and uses kernel functions to handle nonlinearity by transforming data into higher-dimensional spaces [51,52]. By adopting the structural risk minimization (SRM) principle, which is superior in generalization ability compared to the empirical risk minimization (ERM) principle used by neural networks, SVR is capable of capturing structural characteristics from training data, making it effective even with small sample sizes [53,54,55]. This approach enables SVR to capture global trends and complex patterns more effectively, reducing predictive bias. These results emphasize the importance of selecting a model based on the characteristics of the problem and the data. The SVR approach may be more suitable for ruminal protein flow modeling because it reduces bias and effectively captures global trends.

The model developed in this study combines traditionally important input variables with animal-specific information such as DIM, enabling precise and accurate predictions despite challenges like multicollinearity or nonlinear relationships among variables. This success is attributed to the application of machine learning algorithms, which demonstrated superior performance compared to traditional regression-based models, highlighting their potential for practical applications. However, the current model has not yet been validated using data from actual research farms. Future evaluations should focus on utilizing datasets from multiple farms to ensure their reliability and generalizability. Additionally, assessing the model’s adequacy under diverse environmental conditions is essential. Expanding the dataset to include larger and more varied management scenarios would help explore the model’s scalability and robustness. Further, other advanced machine learning techniques, such as gradient boosting, artificial neural networks, or deep learning, should be investigated to determine whether additional accuracy and bias reduction improvements can be achieved.

Building on this foundation, a promising direction for future research could be the development of an HIMM integrating the strengths of AI with mechanistic approaches, such as those employed in NASEM. Mechanistic models, grounded in biological principles, provide theoretical predictions based on nutrient dynamics, while AI excels in handling complex, nonlinear interactions and individual variability. By combining these approaches, an HIMM could leverage mechanistic predictions as foundational inputs, which AI refines to account for individual animal and environmental factors more effectively. This integration may enhance prediction accuracy, particularly when standalone AI or traditional models face limitations, such as under extreme or unique management conditions. If successfully developed, such a model could contribute to advancing intelligent, integrated decision support tools, offering dairy producers precise, scalable solutions to optimize animal productivity and reduce nutrient emissions, aiming to achieve sustainability through high production efficiency. Sustaining optimal rumen function is essential for maximizing microbial efficiency and overall nutrient utilization [13], which are fundamental considerations for integrating AI-driven insights with mechanistic approaches. Integrating AI-driven insights with mechanistic understandings of rumen health could further refine these decision support tools, ensuring that improvements in MP nutrition align with strategies that support microbial balance and digestive efficiency.

## 5. Conclusions

In conclusion, this study demonstrates the potential of machine learning models to enhance the prediction of RUP and MicN flows in dairy cows by incorporating key input variables such as DIM, DMI, and dietary composition. The results highlight the advantages of integrating physiological and dietary factors into prediction models, with machine learning proving effective at managing nonlinear relationships and multicollinearity. While the models showed promising improvements in prediction accuracy, further validation using diverse datasets and real-world farm conditions is required to confirm their reliability and applicability. Future research should focus on scaling these models across varied management scenarios and exploring advanced machine learning algorithms to achieve even greater predictive performance. Additionally, hybrid approaches combining mechanistic models with AI could offer a robust solution for improving accuracy and adaptability. These advancements can enable precision feeding strategies, supporting more tailored feeding programs and promoting sustainable, efficient dairy production systems.

## Figures and Tables

**Table 1:** Descriptive statistics of the whole dataset.

Variables	n	Mean	Median	Min	Max
No. of articles	436	-	-	-	-
No. of treatments	1779	-	-	-	-
No. of animals per treatment	1779	11.2	8.0	2.0	58.0
Animal information					
Days in milk (day)	1397	102.3	99.0	1.0	323.0
Parity	1472	1.9	2.0	1.0	2.0
Body weight (kg)	1481	629.6	624.0	476.0	861.9
Dry matter intake (kg/d)	1779	21.7	21.7	8.8	32.0
Milk yield (kg/d)	1674	33.5	33.9	10.2	58.5
Milk composition (%)					
Lactose	1125	4.7	4.8	3.7	5.7
Fat	1582	3.7	3.7	2.1	5.6
Crude protein	1584	3.1	3.1	2.6	4.1
Dietary chemical composition (%)					
Dry matter	1004	54.0	52.6	13.5	96.3
Organic matter	1045	92.4	92.6	46.1	98.9
Crude protein	1665	16.6	16.6	9.3	29.6
Neutral detergent fiber (NDF)	1520	32.9	32.5	17.6	60.8
Forage NDF	874	23.5	22.8	6.6	54.3
Acidic detergent fiber (ADF)	1222	20.4	20.2	8.8	61.6
Fat	743	3.9	3.7	0.3	8.9
Ash	793	7.4	7.3	1.1	16.5
Non-starch carbohydrate	604	39.4	40.2	16.8	51.2
Starch	931	24.7	25.4	0.2	47.6
N flows at the duodenum (g/d)					
Total N	442	541.7	534.5	200.9	1110.0
Microbial N	577	296.0	276.8	74.0	763.0
Non-ammonia N	550	506.8	504.7	73.0	1078.0
Ammonia N	433	20.6	17.5	1.8	121.9
Non-ammonia, non-microbial N	542	214.6	215.2	33.1	576.0
Digestibility (%)					
Dry matter	664	67.6	67.9	19.7	85.0
Organic matter	772	69.1	69.5	43.8	85.2
Crude protein	694	67.5	67.9	40.3	86.6
NDF	776	49.6	48.6	19.5	84.0
ADF	408	45.3	45.1	18.0	75.3
Fat	225	89.2	89.0	1.0	177.0
Starch	385	94.1	95.8	13.8	99.9
Rumen characteristics					
pH	460	6.1	6.1	5.5	6.9
Total volatile fatty acid (mM)	436	112.9	113.0	11.0	746.4
Ammonia N (mg/dL)	438	13.0	12.3	1.4	40.6

**Table 2:** Descriptive statistics of the dataset for rumen-undegradable protein modeling.

Variables	n	Mean	Median	Min	Max
No. of articles	145	-	-	-	-
No. of treatments	542	-	-	-	-
No. of animals per treatment	542	6.6	4.0	2.0	40.0
Animal information					
Days in milk (day)	376	108.8	97.0	16.0	323.0
Parity	432	1.9	2.0	1.0	2.0
Body weight (kg)	424	607.6	600.0	480.0	788.0
Dry matter intake (kg/d)	542	20.1	19.9	8.8	31.8
Milk yield (kg/d)	448	29.2	29.3	10.2	44.7
Dietary chemical composition (% DM)					
Dry matter (% as-fed)	314	60.5	57.5	15.7	91.5
Organic matter	351	92.2	92.5	46.1	97.7
Crude protein	495	17.2	17.3	9.6	29.6
Neutral detergent fiber	437	32.4	32.3	17.6	50.9
Acidic detergent fiber	393	19.4	19.0	8.8	35.5
Fat	71	4.2	4.0	1.6	6.9
Ash	99	7.1	7.1	5.1	11.7
Non-starch carbohydrate	34	37.1	37.4	22.0	47.9
Starch	231	27.9	28.5	2.5	47.6
N flows at the duodenum (g/d)					
Total N	426	540.4	533.1	200.9	1110.0
Microbial N	542	296.9	276.9	74.0	763.0
Non-ammonia N	542	512.4	505.5	173.0	1078.0
Ammonia N	425	20.3	17.5	1.8	121.9
Non-ammonia, non-microbial N	542	214.6	215.2	33.1	576.0

**Table 3:** Descriptive statistics of the dataset for microbial nitrogen modeling.

Variables	n	Mean	Median	Min	Max
No. of articles	153	-	-	-	-
No. of treatments	577	-	-	-	-
No. of animals per treatment	577	6.5	4.0	2.0	40.0
Animal information					
Days in milk (day)	390	108.3	97.0	16.0	323.0
Parity	454	1.8	2.0	1.0	2.0
Body weight (kg)	459	608.4	600.0	480.0	788.0
Dry matter intake (kg/d)	577	20.0	19.9	8.8	31.8
Milk yield (kg/d)	483	29.0	28.8	10.2	44.7
Dietary chemical composition (% DM)					
Dry matter (% as-fed)	346	59.6	57.1	15.7	93.9
Organic matter	379	92.1	92.5	46.1	97.7
Crude protein	530	17.3	17.3	9.6	29.6
Neutral detergent fiber	464	32.4	32.3	17.6	50.9
Acidic detergent fiber	408	19.5	19.0	8.8	35.5
Fat	90	3.9	3.9	1.6	6.9
Ash	118	7.2	7.2	5.1	11.7
Non-starch carbohydrate	52	37.6	37.6	22.0	47.9
Starch	250	27.1	27.8	2.5	47.6
N flows at the duodenum (g/d)					
Total N	442	541.7	534.5	200.9	1110.0
Microbial N	577	296.0	276.8	74.0	763.0
Non-ammonia N	550	506.8	504.7	73.0	1078.0
Ammonia N	433	20.6	17.5	1.8	121.9
Non-ammonia, non-microbial N	542	214.6	215.2	33.1	576.0

**Table 4:** Adequacy of rumen-undegradable protein (RUP, kg/d) prediction models developed using random forest regression and support vector regression algorithms evaluated on the test set.

	Performance					
			% RMSEP ^1^			
Model	R^2^	RMSEP, kg/d	Mean Bias	Slope Bias	Random Bias	CCC
RFR	0.64	0.352	7.3	3.0	89.8	0.74
SVR	0.38	0.460	4.2	4.1	91.7	0.59

RFR, random forest regression; SVR, support vector regression; NASEM, NRC dairy (2021); RMSEP, root mean square error of prediction; CCC, concordance correlation coefficient. ^1^ There was no statistically significant mean or slope bias (*p* > 0.1).

**Table 5:** Adequacy of newly developed and published models for predicting the rumen-undegradable protein (RUP, kg/d) evaluated using 5-fold cross-validation.

	Performance					
			% RMSEP ^1^			
Model	R^2^	RMSEP, kg/d	Mean Bias	Slope Bias	Random Bias	CCC
RFR	0.60	0.326	4.7 ^1^	5.2 ^2^	90.1	0.71
SVR	0.53	0.349	3.7	1.2	95.1	0.68
NASEM	0.27	0.437	2.9	3.2 ^3^	93.9	0.45

RFR, random forest regression; SVR, support vector regression; NASEM, NRC dairy (2021); RMSEP, root mean square error of prediction; CCC, concordance correlation coefficient. ^1^ Statistically significant mean bias (*p* < 0.05) was shown in 2 out of 5 folds. ^2,3^ Statistically significant slope bias (*p* < 0.05) was shown in 1 out of 5 folds.

**Table 6:** Adequacy of duodenal microbial nitrogen (MicN, g/d) prediction models developed using random forest regression and support vector regression algorithms evaluated on the test set.

	Performance					
			% RMSEP ^1^			
Model	R^2^	RMSEP, g/d	Mean Bias	Slope Bias	Random Bias	CCC
RFR	0.82	49.8	1.1	4.1	94.8	0.89
SVR	0.89	38.6	0.0	4.9	95.0	0.94

RFR, random forest regression; SVR, support vector regression; NASEM, NRC dairy (2021); RMSEP, root mean square error of prediction; CCC, concordance correlation coefficient. ^1^ There was no statistically significant mean or slope bias (*p* > 0.1).

**Table 7:** Adequacy of newly developed models for predicting the duodenal microbial nitrogen (MicN, g/d) evaluated using 5-fold cross-validation.

	Performance					
			% RMSEP ^1^			
Model	R^2^	RMSEP, g/d	Mean Bias	Slope Bias	Random Bias	CCC
RFR	0.69	52.0	4.9	14.1 ^1^	81.1	0.73
SVR	0.76	42.4	1.8	5.6	92.7	0.86
NASEM	0.04	90.7	5.5 ^2^	6.4	88.2	0.13

RFR, random forest regression; SVR, support vector regression; NASEM, NRC dairy (2021); RMSEP, root mean square error of prediction; CCC, concordance correlation coefficient. ^1^ Statistically significant slope bias (*p* < 0.05) was shown in 2 out of 5 folds. ^2^ Statistically significant mean bias (*p* < 0.05) was shown in 1 out of 5 folds.

## Data Availability

The raw data supporting the conclusions of this article will be made available by the authors upon request. The NANP dataset is available at https://animalnutrition.org/.
